# Arginase Activity in *Eisenia andrei* Coelomocytes: Function in the Earthworm Innate Response

**DOI:** 10.3390/ijms22073687

**Published:** 2021-04-01

**Authors:** Joanna Homa, Alina Klosowska, Magdalena Chadzinska

**Affiliations:** Department of Evolutionary Immunology, Institute of Zoology and Biomedical Research, Faculty of Biology, Jagiellonian University, Gronostajowa 9, 30-387 Krakow, Poland; alina.klosowska@gmail.com (A.K.); magdalena.chadzinska@uj.edu.pl (M.C.)

**Keywords:** coelomocytes, innate immune response, arginase, earthworm

## Abstract

Arginase is the manganese metalloenzyme catalyzing the conversion of l-arginine to l-ornithine and urea. In vertebrates, arginase is involved in the immune response, tissue regeneration, and wound healing and is an important marker of alternative anti-inflammatory polarization of macrophages. In invertebrates, data concerning the role of arginase in these processes are very limited. Therefore, in the present study, we focused on the changes in arginase activity in the coelomocytes of *Eisenia andrei*. We studied the effects of lipopolysaccharide (LPS), hydrogen peroxide (H_2_O_2_), heavy metals ions (e.g., Mn^2+^), parasite infection, wound healing, and short-term fasting (5 days) on arginase activity. For the first time in earthworms, we described arginase activity in the coelomocytes and found that it can be up-regulated upon in vitro stimulation with LPS and H_2_O_2_ and in the presence of Mn^2+^ ions. Moreover, arginase activity was also up-regulated in animals in vivo infected with nematodes or experiencing segment amputation, but not in fasting earthworms. Furthermore, we confirmed that the activity of coelomocyte arginase can be suppressed by l-norvaline. Our studies strongly suggest that similarly to the vertebrates, also in the earthworms, coelomocyte arginase is an important element of the immune response and wound healing processes.

## 1. Introduction

Arginase (Arg) is the manganese metalloenzyme catalyzing the conversion of l-arginine to l-ornithine and urea. It has been found in vertebrates and invertebrates, yeast, bacteria, and plants [1]. In the majority of animals, it occurs in two isoforms, from which cytosolic arginase I plays a role in ureagenesis, while mitochondrial arginase II is involved in the biosynthesis of polyamines and in the regulation of inflammatory response [2]. Moreover, as Arg-mediated l-arginine metabolism produces l-ornithine, an important component of collagen synthesis, arginase is involved in tissue regeneration and wound healing [1,3]. Furthermore, animals that metabolize excess nitrogen as urea express a second cytosolic form of arginase named A1 [4].

Interestingly, both in mammals and lower vertebrates, arginase is also involved in macrophage polarization. It was shown that pro-inflammatory cytokines such as tumor necrosis factor (TNF-α) and interferon-gamma (IFN-γ) as well as bacterial lipopolysaccharide (LPS) stimulate classical M1 macrophage polarization. M1 cells have enhanced microbicidal capacity and secrete high levels of pro-inflammatory cytokines (IL-1β, TNF-α, IL-12, and IL-23), chemokines (e.g., CXCL8-11) as well as nitric oxide (NO) and reactive oxygen species (ROS). As a consequence, classically polarized macrophages lead to the effective eradication of the pathogens. In contrast, anti-inflammatory cytokines (IL-4, IL-13, and IL-10) polarize macrophages to the alternative anti-inflammatory M2 phenotype. M2 cells down-regulate the inflammatory response to avoid host tissue damage. This comprises enhancement of phagocytosis, promotion of tissue repair, and elimination of parasites [5,6]. A typical difference between M1 and M2 macrophages is their l-arginine metabolism, involving either inducible nitric oxide synthase (iNOS) in classically-activated macrophages or arginase in alternatively-activated macrophages [5]. An upregulation of arginase expression and activity in M2 macrophages induced the formation of and polyamines (e.g., putrescine, spermidine, and spermine), which support cell growth, motility, and survival, as well as maintenance of chromatin conformation and free-radical scavenging. Moreover, polyamines are essential for collagen synthesis [7,8].

In invertebrates, three enzymes representing different arginine metabolic pathways (nitric oxide synthase (NOS), arginase, and agmatinase) were identified and well characterized. For example, in sea cucumber (*Apostichopus japonicus*), all three enzymes were found in intestine, tentacle, respiratory tress, and coelomocytes [9]. Moreover, bacterial challenge or LPS-treatment significantly up-regulated gene expression of AjNOS, but down-regulated Ajarginase, induced NO production and suppressed arginase activity.

In earthworms, which are ureotelic organisms, arginase was, for the first time, described by Cohen and Lewis [10]. In earthworms, arginase is mainly present in the intestinal tissue, which is related to the enzymatic synthesis of citrulline from l-ornithine, ammonia bicarbonate, and adenosine triphosphate, which takes place in the soluble fraction of gut tissue [10,11,12]. According to our understanding, there are no data describing the expression and function of arginase in the earthworm immune system. Here, it is important to mention that earthworms have very efficiently functioning cellular and humoral immune mechanisms that allow them to survive in their natural, rich in pathogens and environment. Their main immunocompetent cells are coelomocytes, which have been divided into three different subpopulations: chloragocytes (eleocytes), hyaline amebocytes, and granular amebocytes [13]. Both hyaline and granular amebocytes are phagocytic cells and similarly, to mammalian macrophages, migrate to the site of infection, engulf or encapsulate pathogens and ROS and NO [14,15,16,17,18]. In contrast, eleocytes are mainly involved in nutrition, excretion (e.g., fetidins and lysenin), and the synthesis of cytotoxic and antibacterial molecules [19,20,21].

The aim of the present study was to determine the role of coelomocyte arginase in the immune response against bacteria, parasites, in the process of wound healing as well as during fasting, in earthworm *Eienia andrei*.

## 2. Results

### 2.1. Effects of In Vitro Coelomocyte Stimulation on Arginase Activity

**Figure 1:**
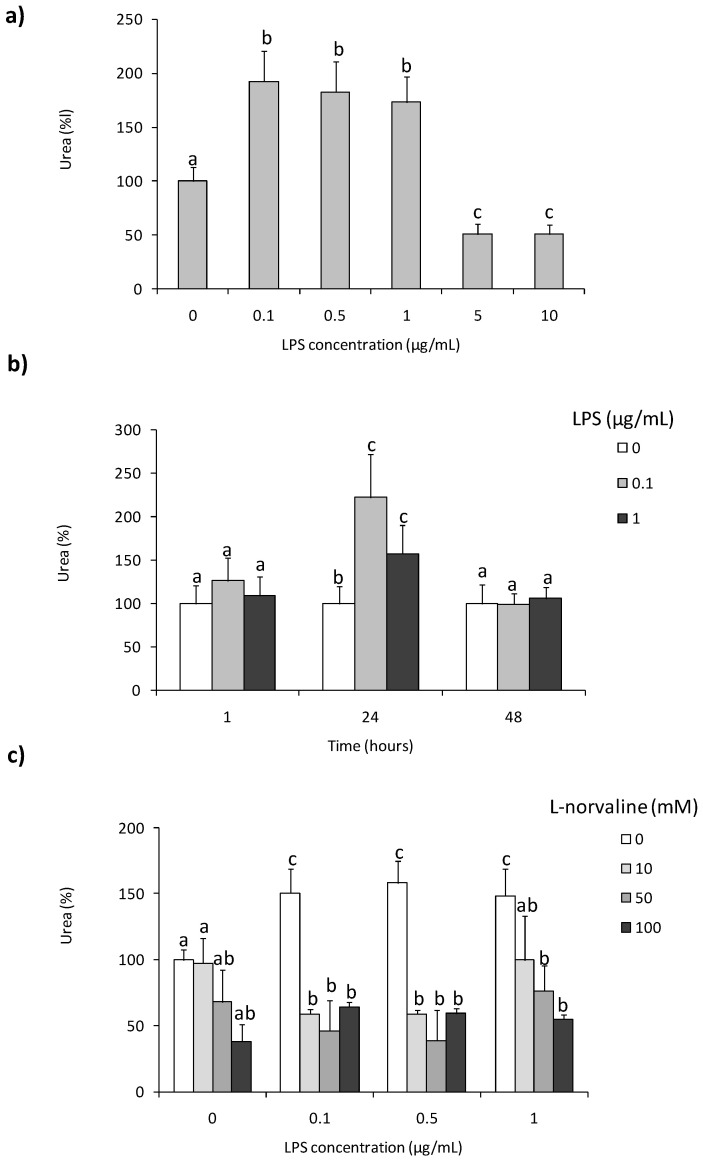
Effects of in vitro stimulation with lipopolysaccharide on arginase activity in *Eisenia andrei* coelomocytes. Cells were in vitro treated with lipopolysaccharide (LPS) (0–10 µg/mL) for 24 h (**a**) or with LPS (0–1 µg/mL) for 1, 24, or 48 h (**b**). Some cells 1 h before stimulation were treated with arginase activity inhibitor (l-norvaline, 10–100 mM) (**c**). Arginase activity was determined as urea content and showed as % of control (arginase activity in unstimulated cells = 100%). Mean + standard errors (SE), n = 8–16. Mean values that are significantly different are not assigned a common letter (e.g., a vs. b or b vs. c) according to one-way analysis of variance (ANOVA) (at *p* < 0.05).

To assess whether LPS was able to stimulate arginase activity in coelomocytes, cells were treated with different concentrations of this immunostimulant. Increased arginase activity was observed in coelomocytes stimulated 24 h with LPS (0.1–1 µg/mL), whereas LPS in concentrations of 5 and 10 µg/mL decreased arginase activity Figure 1a,b. Similar changes were not observed at 1 and 48 h of incubation/stimulation. In both time points, arginase activity was on a low control level Figure 1b. Arginase inhibitor–l-norvaline did not affect arginase activity in controlling unstimulated cells while inhibiting LPS-induced up-regulation of its activity. In cells stimulated with 0.1 and 0.5 µg/mL of LPS, the inhibitory effect of l-norvaline was observed for all used concentrations of the inhibitor while in cells treated with a high concentration of LPS (1 µg/mL), inhibition was observed for two concentrations of l-norvaline (50 and 100 mM) Figure 1c.

**Figure 2:**
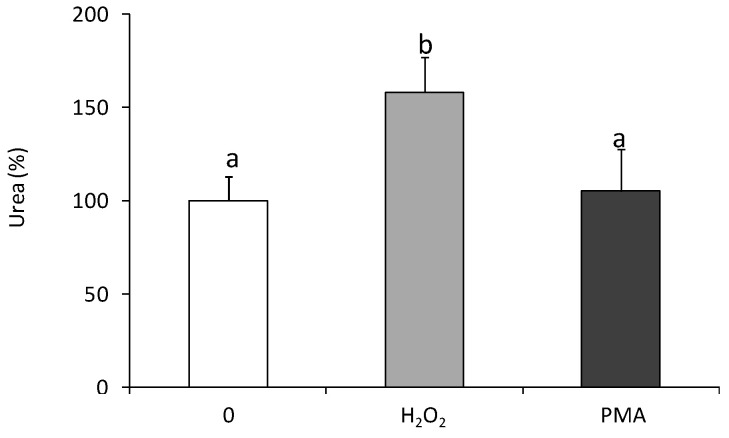
Effects of in vitro stimulation with hydrogen peroxide or phorbol ester on arginase activity in *Eisenia andrei* coelomocytes. Cells were in vitro treated for 24 h with H_2_O_2_ (50 µM), phorbol 12-myristate 13-acetate, PMA (0.1 µg), or with culture medium (0). Arginase activity was determined as urea content and showed as % of control (arginase activity in unstimulated cells = 100%). Mean + SE, n = 6–9. Mean values that are significantly different are not assigned a common letter (e.g., a vs. b or b vs. c) according to one-way analysis of variance (ANOVA) (at *p* < 0.05).

Moreover, statistically significant up-regulation of arginase activity was found in coelomocytes treated for 24 h with H_2_O_2_ but not in cells stimulated with phorbol 12-myristate 13-acetate (PMA) (Figure 2).

### 2.2. Effects of In Vitro Heavy Metal Treatment of Coelomocytes on Arginase Activity

**Figure 3:**
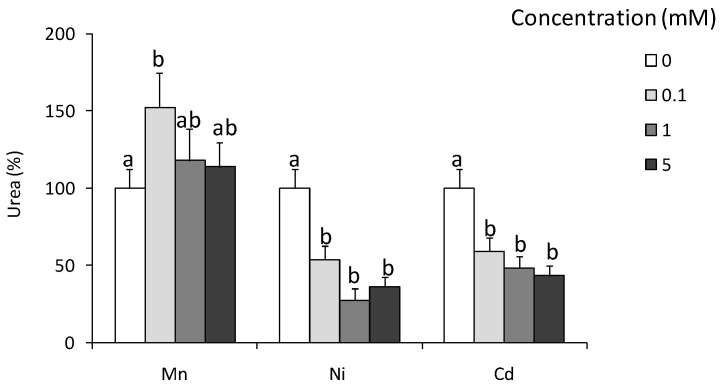
Effects of in vitro treatment with heavy metals on arginase activity in *Eisenia andrei* coelomocytes. Cells were in vitro treated for 24 h with different concentrations of Ni, Cd, and Mn chloride (0.1, 1, or 5 mM). Arginase activity was determined as urea content and showed as % of control (arginase activity in unstimulated cells = 100%). Mean + SE, *n* = 4–10. Mean values that are significantly different are not assigned a common letter (e.g., a vs. b or b vs. c) according to one-way analysis of variance (ANOVA) (at *p* < 0.05).

Mn^2+^ ions in concentration 0.1 mM increased the arginase activity by 40–50%, while Ni^2+^ and Cd^2+^ in all tested concentrations down-regulated arginase activity (Figure 3).

### 2.3. In Vivo Effects of LPS

**Figure 4:**
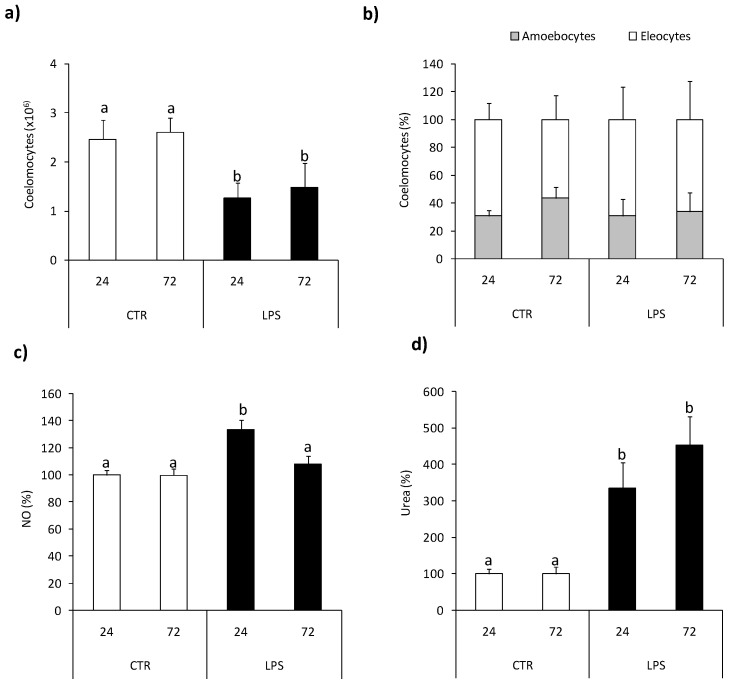
Effects of in vivo stimulation with lipopolysaccharide on arginase activity and nitric oxide production in *Eisenia andrei* coelomocytes. Coelomocytes were collected 24 or 72 h post earthworms injection with 0.9% sodium chloride (CTR) or with LPS (1 mg/mL). Number (**a**) and composition (**b**) of coelomocytes was counted. Nitric oxide (NO) production (**c**) and arginase activity (**d**) are showed as % of control (NO production and arginase activity in coelomocytes of CTR animals = 100%). Mean + SE, n = 8. Mean values that are significantly different are not assigned a common letter (e.g., a vs. b or b vs. c) according to one-way analysis of variance (ANOVA) (at *p* < 0.05).

At 24 and 72 h post-LPS-injection, a significant reduction in the number of coelomocytes was observed Figure 4a; however, such treatment did not change coelomocyte composition Figure 4b. At both time points, LPS-treatment enhanced arginase activity Figure 4d while LPS-induced up-regulation of NO production was observed at 24 h post-injection only Figure 4c.

### 2.4. In Vivo Effects of Nematode Infection

**Figure 5:**
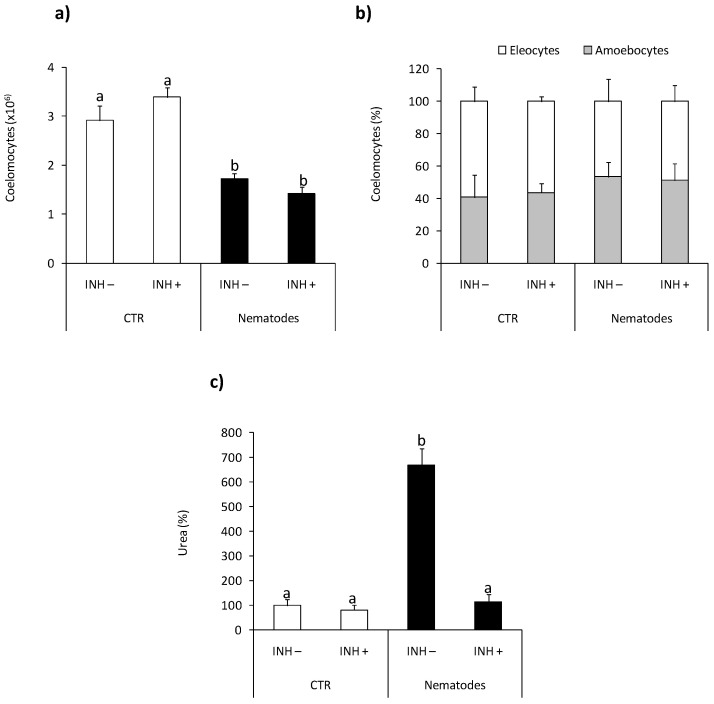
Effects of in vivo nematode infection on arginase activity in *Eisenia andrei* coelomocytes. Coelomocytes were collected 24 h post earthworms injection with 0.9% sodium chloride (CTR) or with nematodes (*S. feltiae*, 1000–1500 nematodes/mL). Prior to stimulation, some earthworms were injected for 1 h with arginase inhibitor l-norvaline (100 mM, INH+) or sodium chloride (INH–). The number (**a**) and composition (**b**) of coelomocytes were counted. Arginase activity was determined as urea content and showed as % of control (arginase activity in coelomocytes of CTR animals = 100%) (**c**). Mean values that are significantly different are not assigned a common letter (e.g., a vs. b or b vs. c) according to one-way analysis of variance (ANOVA) (at *p* < 0.05).

At 24 h post nematode infection reduced number of coelomocytes retrieved from the coelomic earthworm cavity was observed compared to control animals Figure 5a, while infection did not affect coelomocyte composition Figure 5b. Neither the number nor composition of coelomocytes was changed upon l-norvaline pre-treatment Figure 5a,b.

At the same time point, post-injection coelomocytes from nematode-infected earthworms showed significantly higher arginase activity than animals injected with saline. Nematode-induced up-regulation of arginase activity was not observed in animals pretreated with l-norvaline Figure 5c.

### 2.5. Effects of Tissue Injury

**Figure 6:**
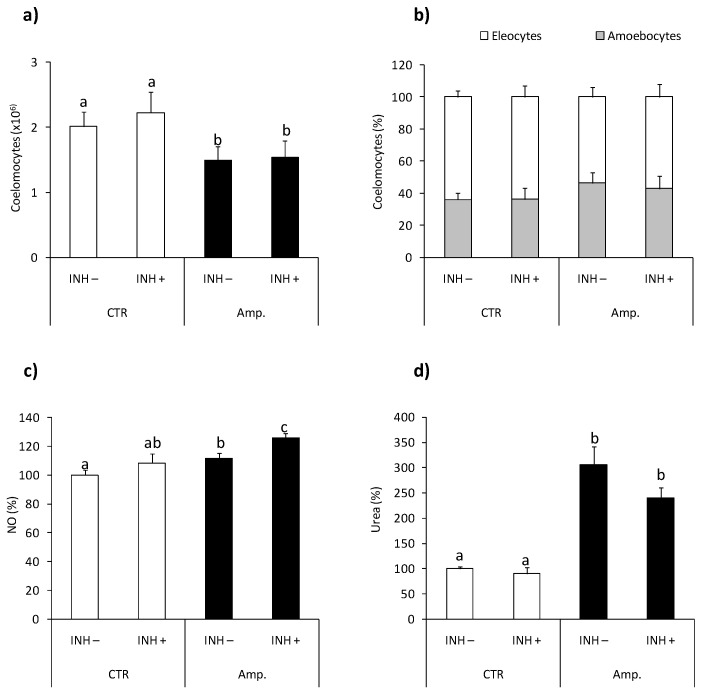
Effects of segment amputation on arginase activity and nitric oxide production in *Eisenia andrei* coelomocytes. Coelomocytes were collected from control animals (CTR) or from animals 72 h post-amputation of 5 posterior segments (Amp.). Earthworms were injected for 1 h with arginase inhibitor l-norvaline (100 mM, INH+) or with 0.9%NaCl (INH–). The number (**a**) and composition (**b**) of coelomocytes were counted. Nitric oxide (NO) production (**c**) and arginase activity (**d**) are showed as % of control (NO production and arginase activity in coelomocytes of CTR animals = 100%). Mean values that are significantly different are not assigned a common letter (e.g., a vs. b or b vs. c) according to one-way analysis of variance (ANOVA) (at *p* < 0.05).

The numbers of coelomocytes retrieved from earthworms with amputated (Amp.) posterior segments were lower in comparison with controls animals Figure 6a, while tissue injury did not change coelomocyte composition Figure 6b. Neither the number nor composition of coelomocytes was changed upon l-norvaline injection Figure 6a,b. Coelomocytes retrieved from animals with amputated segments show slightly but significantly up-regulated NO production compared to cells from control animals Figure 6c. Coelomocytes from Amp. earthworms also had higher arginase activity Figure 6d than control worms. l-norvaline slightly but not significantly (*p* = 0.0579) reduced arginase activity in coelomocytes of animals with amputated segments while it significantly increased the level of NO released from coelomocytes retrieved from injured animals Figure 6c,d.

### 2.6. Effect of Fasting

**Figure 7:**
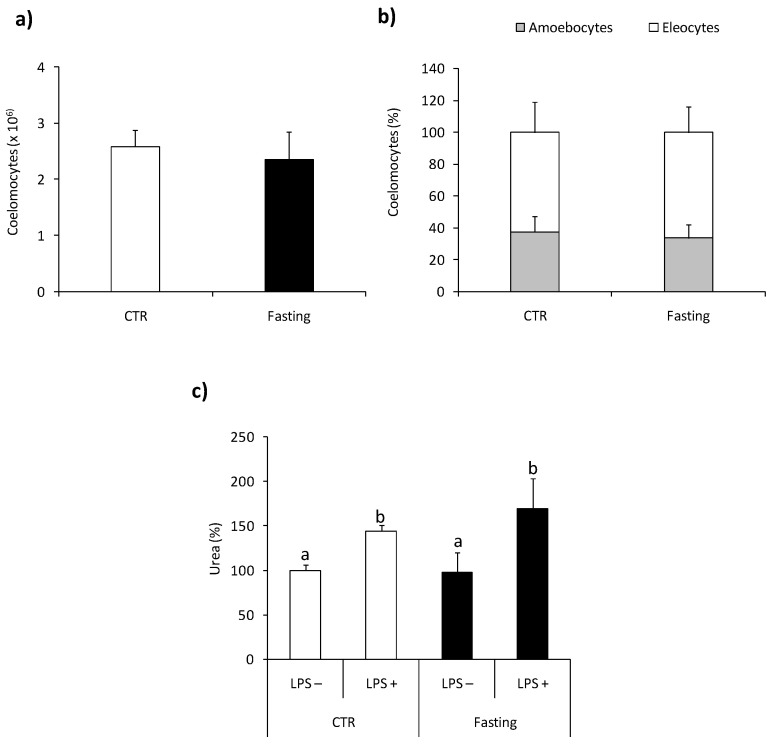
Effects of short-term fasting on arginaseactivity in *Eisenia andrei* coelomocytes. Coelomocytes were collected from earthworms kept in soil (CTR) or exposed for 5 days to filter paper soaked with distilled water (Fasting). The number (**a**) and composition (**b**) of coelomocytes were counted. Arginase activity (**c**) was measured in coelomocytes incubated for 24 h with control medium (LPS–) or with LPS (1 µg/mL, LPS+) and is shown as % of control (arginase activity in LPS-coelomocytes of CTR animals = 100%). Mean + SE, *n* = 10. Mean values that are significantly different are not assigned a common letter (e.g., a vs. b or b vs. c) according to one-way analysis of variance (ANOVA) (at *p* < 0.05).

Fasting (five days) did not change the number and composition of coelomocytes Figure 7a,b. Moreover, arginase activity in coelomocytes from control and fasting animals was similar and exhibited similar sensitivity to ex vivo LPS stimulation Figure 7c.

## 3. Discussion

In the present studies, we focused on the effects of in vitro and in vivo stimulation of immune response on arginase activity in earthworm coelomocytes. According to our understanding, we provide the first evidence that arginase is fully operating in *Eisenia andrei* coelomocytes and that both in vitro and in vivo immunostimulation induced arginase activity. We found that both in vitro and in vivo lipopolysaccharide stimulates arginase activity in earthworm coelomocytes, and in the case of in vitro study, this effect was dependent on the time of stimulation and LPS concentration and decreased by l-norvaline pre-treatment. In vivo, the injection of earthworms with LPS decreased the number of coelomocytes and up-regulated the release of NO and arginase activity.

It is known that in vertebrates, lipopolysaccharide is capable of concurrently stimulating nitric oxide production and arginase activity in macrophages [22,23]. For example, LPS-induced up-regulation of arginase activity was observed in vitro in the cell line of mouse macrophages RAW264.7 [2,22], rat alveolar macrophages [24], or the human monocyte cell line THP-1 [25]. Also, in fish macrophages, LPS induced Arg expression and activity, as found in our previous studies [26,27]. Data concerning arginase expression and activity in the immunocompetent cells of invertebrates are very limited. Previously, the changes in the arginase activity upon stimulation with LPS or Schistosomamansoni sporocysts were found in sea cucumber (*Apostichopus japonicus)* [9,28] and in snails (*Biomphalaria glabrata*) [29]. Moreover, the previous studies of sea cucumber *A. japonicus* showed that Gram-negative bacteria *Vibrio splendidus* challenged coelomocytes and intestine, and LPS-exposed primary coelomocytes, significantly increased *NOS* expression, which was followed by down-regulation of *Arginase* and *Agmatinase* transcripts [9]. Such phenomenon was observed when the dose of pathogen measured as a multiplicity of infection was around 10, while higher pathogen dose (multiplicity of infection, MOI = 100) increased arginase activity and decreased NO production [9]. The study of the mechanism regulating arginase and agmatinase gene expression indicated that both Ajagmatinase and Ajarginase promoter activities were significantly activated by LPS and l-arginine challenge [28]. In turn, in *B. glabrata* snail hemocytes, sporocyst stimulation upregulated synthesis of arginase 1, a competitor of nitric oxide synthetase, and inhibitor of larval-killing NO production [29].

LPS or bacterial challenge increases in arginase expression and/or activity contribute to the earthworm immune response’s deactivation and prevents the inflammation-induced destruction of host cells and tissues [30]. For example, the results obtained from the non-transformed intestinal cells from the IPEC-J2 line show that the Arg-1 signaling pathway is partly responsible for anti-inflammatory and antioxidant effects and protects host cells from LPS-induced exaggerated inflammatory response and oxidative stress [31].

To confirm that the enzyme found in earthworm coelomocytes is arginase, an l-norvaline inhibitor was used. This inhibitor acts directly on the enzyme by mimicking ornithine structure and indirectly inhibiting ornithine transcarbamylase [32,33] and succinate synthetase [32,33,34]. In our studies, l-norvaline suppressed enzyme activity in coelomocytes stimulated by LPS in all used inhibitor concentrations, indicating that *E. coli* lipopolysaccharide indeed activated arginase activity in earthworm coelomocytes. Our results are similar to results conducted on the cell lines of mouse macrophages J774A.1, where l-norvaline in a concentration of 10 mM inhibited arginase activity, reducing urea production by 50% [33]. We demonstrated that l-norvaline has a direct inhibitory effect on arginase function. The specificity of l-norvaline in arginase inhibition has been reported to be one of the most effective arginase inhibitors in various preparations [33]. In our experiments, after 72 h post-l-norvaline injection, there was no effect on arginase inhibition in coelomocytes, whereas, after 24 h, arginase activity significantly decreased. Similar relationships were also found for an NOS inhibitor l-NAME (N^ω^-nitro-l-arginine methyl ester), where the single injection of L-NAME resulted in an inhibition of NO only during the first 24 h infection [35].

In the present in vitro studies, increased arginase activity was also observed in coelomocytes treated with H_2_O_2_, but not in cells treated with PMA. Previous studies on porcine arteries also showed that exposure to hydrogen peroxide increased arginase activity [36]. Moreover, it was reported that the oxidative species, e.g., H_2_O_2_, increased arginase activity in endothelial cells and that this effect was connected with the protein kinase PKC-mediated activation of RhoA/Rho kinase pathway [37]. In contrast to our study, PMA-induced THP-1 monocytes differentiation into macrophages was accompanied by an increased Arg-II expression without detectable upregulation of iNOS or Arg-I [25].

Interestingly, but not surprising, in coelomocytesin vitro treated with Mn^2+^ ions, increased arginase activity was observed, while similar concentrations of Ni^2+^ and Cd^2+^ decreased its activity. It has to be mentioned that arginase is a metalloenzyme in which manganese acts as a cofactor as well as an activator and that required Mn^2+^ as an essential element to exhibit maximal enzyme activity [1]; however, the amount essential for optimal activity of the enzyme varies depends on arginase isoforms and location (e.g., kidney and liver) [38]. For example, it was shown that the cationic rat liver enzyme inactivated by EDTA-treatment (ethylenediaminetetraacetic acid) was fully restored by Mn^2+^, whereas Cd^2+^, Ni^2+^, and Co^2+^ induced effects were smaller [39].

Next to LPS-induced arginase activation, we also observed its higher activity in coelomocytes retrieved from earthworms in vivo infected with nematodes, which suggests its involvement in the anti-parasite response. It was demonstrated that *S. feltiae* are mainly infective for insects; however, it was previously found that earthworms can increase the distribution of nematodes in the soil by transporting them through the digestive tract [40,41]. It is worth mentioning that such conditions decreased the number of coelomocytes, which most probably were recruited for parasite encapsulation/brown body formation. Such a phenomenon was recently observed and both in vivo (upon injection) and in vitro nematodes activated coelomocytes of *E. andrei* and induced the formation of “brown bodies” [42] (Figure S1). Previously, similar coelomocyte activation was also observed in *E. andrei* injected with *Aeromonas hydrophila* [43]. Interestingly, parasite infection did not affect coelomocyte composition, and the percentage of amoebocytes and eleocytes in the whole population of coelomocytes did not change. Therefore, it can be concluded that, when induced by parasite infection, the arginase activity is not due to changes in the ratio of amoebocytes and eleocytes. Previously, arginase involvement in the anti-parasite response was described during *Trypanosoma cruzi* [44], *T. brucei*, *Leishmania*, and *Schistosoma mansoni* infection [45]. Stempin and co-workers [30] found that induction of arginase-1 favors *T. cruzi* persistence in the host [30]. Moreover, in *T. cruzi* infected rats, high arginase-1 activity inhibited NO production in peripheral blood monocytes [46]. As mentioned before in inflammatory cells, l-arginine can be metabolized either by nitric oxide synthase (NOS) to NO and citrulline, or by arginase to ornithine and urea [34]. Most probably, some parasites protect themselves against the toxic effects of NO by inducing high levels of arginase in the host cells. In this way, they deplete l-arginine and reduce the synthesis of toxic NO [35].

Interestingly, the arginase-mediated metabolism of l-arginine is an important source of local ornithine, a proline precursor crucial for collagen synthesis, and therefore the important role of arginase in the process of wound healing and tissue regeneration was discovered [47]. In our experiments, amputation of posterior segments of earthworms reduced the number of coelomocytes, most probably recruited to the site of wound healing, and most importantly, activated coelomocytes to produce NO and increase arginase activity. Although, in this case, we did not find significant l-norvaline-induced inhibition of arginase activity; l-norvaline increased slightly but significantly NO production. It may suggest that also in earthworm coelomocyte NO synthase and arginase compete for the availability of l-arginine. Recently, Bodó and co-authors [48] found that in *E. andrei*, amputation of the first or last five segments induced very fast healing where undifferentiated cells covered the wound’s surface. In our experiments, after amputation, similar rapid (few days) healing processes were observed.

Previously, upregulation of arginase activity was found during skin wound healing in rats (3–14 days post wounding). In this work, iNOS activity was significantly upregulated in the wound tissues only at early time points (12 h–3 days post wounding), and its activity gradually decreased after the third-day post wounding [49]. It has to be mentioned that, in vertebrates, many cells such as macrophages, keratinocytes, and endothelial cells synthesize NO during acute wound healing. In contrast, arginase is synthesized mainly in macrophages and thought to be released into the wound environment by cell death [50]. An analysis of the limb regeneration process in axolotl indicated that macrophages, along with the simultaneous induction of pro- and anti-inflammatory cytokines, are crucial in early response to injury and limb regeneration. Moreover, the authors found that macrophage depletion did not affect epithelial wound closure after limb amputation while it caused excessive fibroplasia, collagen deposition, and a complete block in blastemal formation [51].

Among the many functions of arginase, its activity may be associated with the process of starvation or fasting. The results obtained in experiments with fasting animals differ between organisms and the time of fasting. Studies of the vertebrates, including pigs, have shown that fasting of four and eight days increases hepatic arginase activity, as measured by urea production [52]. In rainbow trout (*Oncorhynchus mykiss*), a twofold increase in hepatic arginase II mRNA expression, but not arginase I, was observed after six weeks of fasting [53]. Also, in earthworm *Lumbricus terrestris*, after 14 days and longer time (24–30 days) of fasting, increased arginase activity was observed in the intestinal tissue [10]. In contrast to previous studies on *L. terrestris*, five days fasting of *E. andrei* did not change arginase activity in coelomocytes. However, studies on *L. terrestris* measured arginase activity in the intestinal tissue, while in the present studies, we specifically study coelomocytes. Furthermore, it was shown that, in the intestinal tissue of *L. terrestris*, arginase activity was up-regulated only after a long time of fasting [10]. In our experiments in coelomocytes derived from earthworms fasted by five days, the arginase activity was similar to that in not fasted control groups. Moreover, additional stimulation with LPS caused a similar reaction in coelomocytes derived from control and five-day fasted earthworms. The effect of short-term starvation on other earthworms *Eisenia veneta*, showed small metabolic changes for the first five days of fasting, with clear differences at six and seven days. These differences included increases in glutamate, citrate, aspartate, and isoleucine, and decreases in lysine, isoleucine, and threonine. [54].

In summary, in the present study, for the first time in earthworms, we describedarginase activity in coelomocytes and found that it can be up-regulated upon immunostimulation/infection, in the presence of Mn^2+^ ions and during wound healing upon segment amputation. Furthermore, we confirmed that l-norvaline can suppress the activity of coelomocyte arginase. Similar changes of arginase activity in immunocompetent earthworm cells and vertebrate leukocytes strongly suggested that the role of arginase in earthworms is not limited to participation in the urea cycle, and that it is an important element of the invertebrate immune response. However, the hypothesis suggesting that earthworm coelomocytes may polarize towards pro-inflammatory (with high iNOS activity) or anti-inflammatory (with high arginase activity) cells depending on the type of infection or tissue damage requires further intensive research.

## 4. Materials and Methods

### 4.1. Animals

Adult (clitellate) earthworms *Eienia andrei* (Sav.) (0.41–0.83 g body weight) from the stock breeding maintained in the Institute of Zoology and Biomedical Research, Jagiellonian University, Krakow, Poland, were kept in controlled laboratory conditions (21 ± 1 °C; 12:12 LD) in commercial metal-free soil (PPUH Biovita, Tenczynek, Poland). Animals were fed twice a week with flour.

### 4.2. In Vitro Experiments

The earthworms were stimulated for 1 min with a 4.5 V electric current to expel coelomic fluid with coelomocytes through the dorsal pores according to the procedure described previously [55,56]. Cells were collected into 0.05 mM Sörensen buffer (Na_2_HPO_4_-KH_2_PO_4_, POCh, Gliwice, Poland), pH 7.4 [57]. Obtained cells were counted with a hemocytometer and resuspended in Sörensen buffer to a density of 1 × 10^6^ cells per mL.

Cells were treated with lipopolysaccharide from *Escherichia coli* 0111:B4 l (LPS, 0.1–10 μg/mL), phorbol 12-myristate 13-acetate (PMA, 0.1 μg/mL), hydrogen peroxide (H_2_O_2_, 50 μM), or with heavy metals: manganese chloride (Mn^2+^, 0.1–5 mM), cadmium chloride (Cd^2+^, 0.1–5 mM), or nickel chloride (Ni^2+^, 0.1–5 mM) for 1, 24, or 48 h. Additionally, it was checked that the solvent control, DMSO (use only for PMA stock resuspension), in the concentration used for PMA did not affect cell activity. We used Sörensen buffer, and control cells were treated with Sörensen buffer for all reagents as solvent.

Additionally, to verify the direct involvement of earthworm arginase in l-arginine metabolism 1 h before stimulation some coelomocytes were pretreated with arginase inhibitor, l-norvaline (10, 50, and 100 mM). All chemicals were obtained from Sigma-Aldrich, St. Louis, MO, USA.

### 4.3. In Vivo Experiments

#### 4.3.1. In Vivo Immunostimulation

Before stimulation, earthworms were rinsed with water and injected with 20 µL of stimulant solution into the coelomic cavity (1 cm behind the clitellum). Earthworms were stimulated either with: LPS (1 mg/mL of 0.9% sodium chloride solution, Baxter Terpol, Poland) or nematodes *Steinernema feltiae.* Infective juvenile parasites, a commercial strain of *S. feltie*, e-nema, was kindly provided by R.-U. Ehlers and harvested by filtration. Next, nematodes were washed three times in 0.9%NaCl, heat killed (65 °C), and prepared as fresh (no fixed) solution at a concentration of 1000–1500 nematodes/mL of sodium chloride.

Some earthworms, 1 h before immunostimulation, were injected with 20 µL of l-norvaline (100 mM). Control (CTR) animals were injected with 0.9%NaCl.

After injection, earthworms were placed individually in 15 mL vials filled with filter paper that was soaked with water [15]. Subsequently, coelomocytes were collected at 24 h and/or 72 h post immunostimulation. Cells were counted with a hemocytometer and their composition was evaluated based on the morphology of amoebocytes (A) and eleocytes (E) [58].

#### 4.3.2. Tissue Injury and Animal Fasting

To verify arginase involvement in tissue regeneration process, five posterior segments were amputated from the animals Figure S2. After surgery, animals were placed individually for 3 days in 15 mL vials filled with filter paper soaked with water. Some animals 1 h before injury were injected with 20 µL of l-norvaline (100 mM). Analysis was performed in four independent experiments in duplicates for controls and for three for the amputation group. Earthworm coelomocytes were retrieved and counted as described in Section 4.3.1.

To investigate effect of fasting on arginase activity, earthworms were placed in boxes with filter paper soaked with water (3 individuals per box) for 5 days. After the fasting period, earthworm coelomocytes were retrieved and counted as described in Section 4.3.1. Some cells were left untreated (-LPS) while some were ex vivo stimulated for 24 h with LPS (1 µg/mL, +LPS).

### 4.4. Coelomocyte Activity

To measure activity of iNOS and arginase, coelomocyte number was always adjusted to a density of 1 × 10^6^ cells per ml of Sörensen buffer.

#### 4.4.1. Arginase Activity

Arginase activity was measured as described by Corraliza et al. [59]. Cells were lysed in 50 μL of 0.1% Triton X-100 containing 5 μg of pepstatin (Sigma-Aldrich, St. Louis, MO, USA), 5 μg of aprotinin (Sigma-Aldrich, St. Louis, MO, USA), and 5 μg of antipain (Sigma-Aldrich, St. Louis, MO, USA), at room temperature for 30 min. Then, 35 microliters of 10 mM MnCl_2_ (Sigma-Aldrich, St. Louis, MO, USA) and 50 mM Tris–HCl (pH 7.5) (Tris, Biorad, USA; HCl, POCH, Gliwice, Poland) was added, and the mixture was incubated for 10 min at 55 °C. 50 μL of 0.5 M l-arginine (pH 9.7) (Sigma-Aldrich, St. Louis, MO, USA) was added to 50 μL of this activated lysate, and incubated for 1 h at 37 °C. The reaction was stopped with 400 μL of the mixture containing H_2_SO_4_ (POCH, Gliwice, Poland), H_3_PO_4_ (Chempur, Poland), and H_2_O (1:3:7), then 25 μL of 9% α-isonitrosopropiophenone (Sigma-Aldrich, St. Louis, MO, USA) in 100% ethanol (99.8%, POCH, Gliwice, Poland). The sample was incubated at 100 °C (45 min) and cooled in the dark (10 min). Absorbance (O.D.) was read at 540 nm, and arginase activity was calculated to compare to the urea standard curve [26].

#### 4.4.2. Nitric Oxide Release

Nitrite/nitrate production, an indicator of nitric oxide synthesis, was measured in cell culture supernatants as described previously [60]. Following incubation 100 μL of cell culture supernatant was added to 100 μL 1% (*w*/*v*) sulfanilamide in 2.5% (*v*/*v*) phosphoric acid and 100 μL of 0.1% (*w*/*v*) *N*-naphthyl-ethylenediamine in 2.5% (*v*/*v*) phosphoric acid (all from Sigma–Aldrich, St. Louis, MO, USA). The O.D. was read at 540 nm.

### 4.5. Data Analysis and Statistics

Results are expressed as means ± standard errors (SE). Statistically significant differences between means were evaluated using a one-way ANOVA test. The level of significance was established at *p* < 0.05.

## Data Availability

The data presented in this study are available in the article.

## Supplementary Materials

The following are available online at https://www.mdpi.com/article/10.3390/ijms22073687/s1.
