# Supplementary material for: Arginase Activity in Eisenia andrei Coelomocytes: Function in the Earthworm Innate Response
Source: Int J Mol Sci. 2021 Apr 1;22(7):3687. doi: 10.3390/ijms22073687 (PMC8037997; doi:10.3390/ijms22073687)
Supplement: Supplementary file 1 [file ijms-22-03687-s001.pdf]

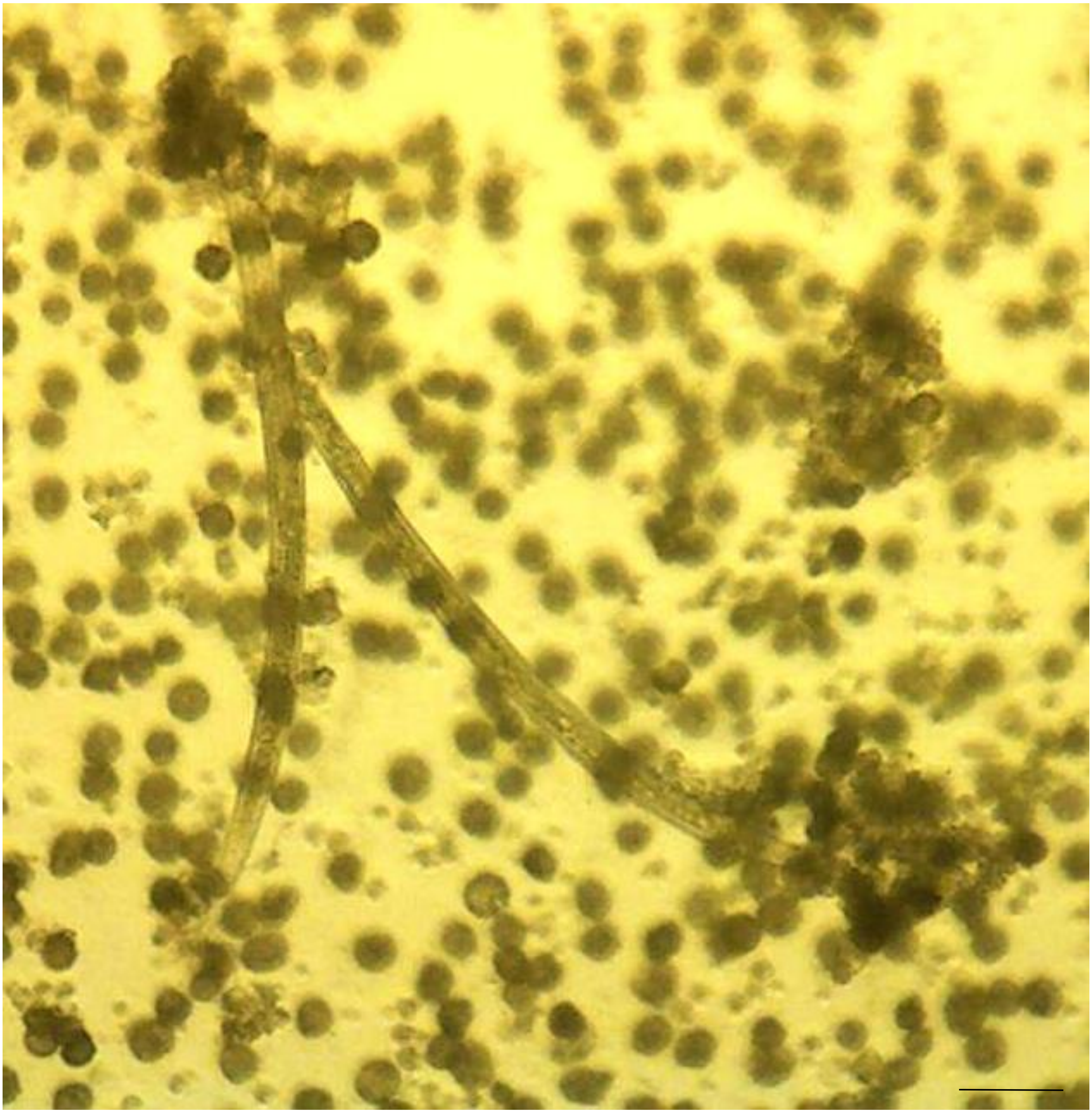

Figure S1. Representative microphotograph of *E. andrei* coelomocytes encapsulating *S. feltiae* nematodes in *in vitro* condition. Scale bar 50 µm.

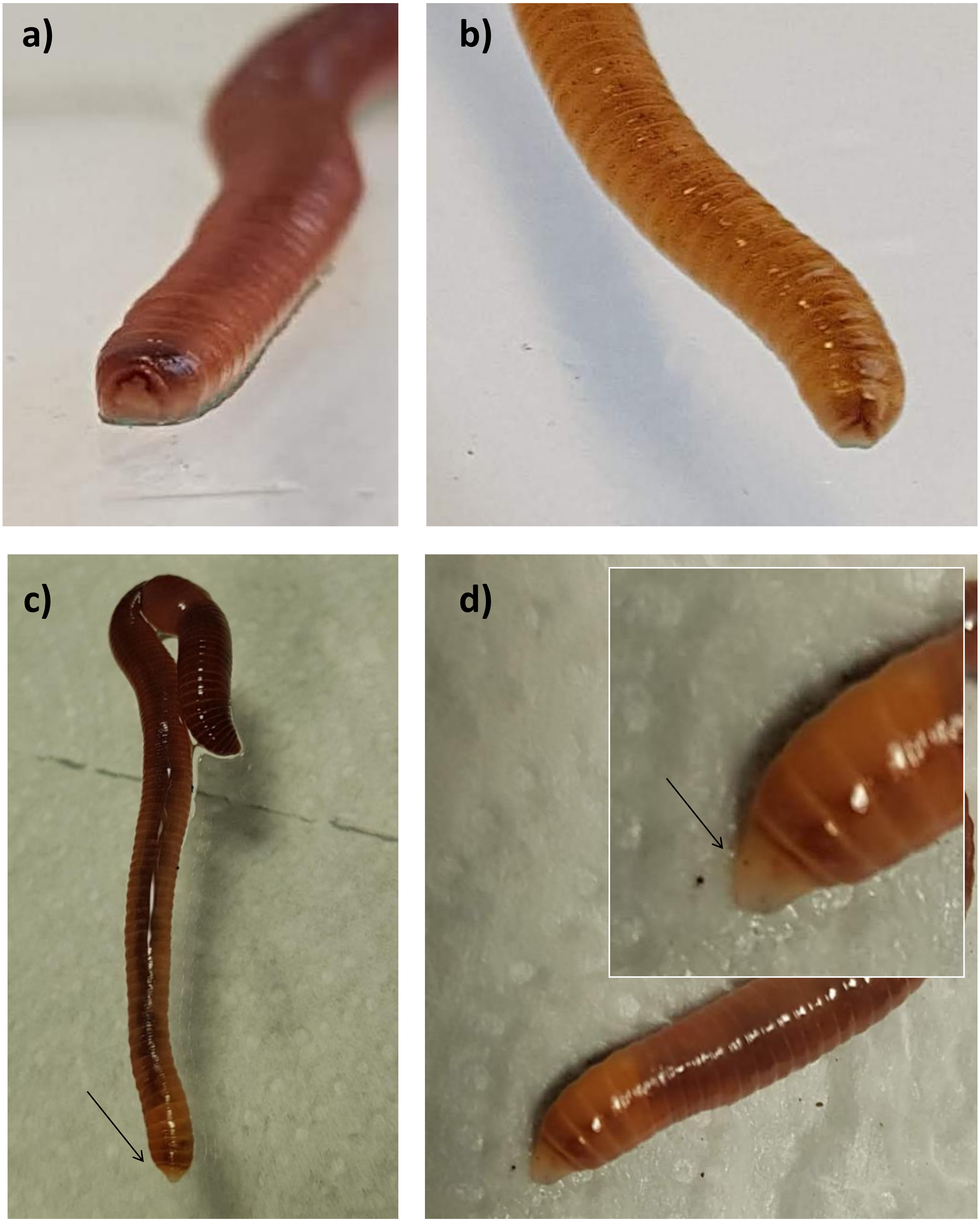

Figure S2. Representative pictures of earthworms: a-b) just after amputation of the last 5 segments of the body, and c-d) after end of experimental procedure, 14 days since amputation (animals were kept in soil in individuals boxes) with clearly visible regenerated part of body (arrow).
